# ADDRESSING THE NEEDS OF AFRICAN AMERICAN BEREAVED DEMENTIA CAREGIVERS

**DOI:** 10.1093/geroni/igad104.0028

**Published:** 2023-12-21

**Authors:** Zachary Baker, Katherine Supiano

## Abstract

Within the next 10 years there will be at least 9 million NEWLY Bereaved Dementia Caregivers, disproportionately affecting African American families due, in part, to dementia prevalence that is twice that of White counterparts. Up to 26% of Bereaved Dementia Caregivers experience pathologically prolonged or exacerbated grief, often referred to as complicated grief or prolonged grief disorder: 2.5x the rate of other chronic disease care contexts, like cancer. The present project used an asset-based approach to assess the needs and resources of African American Bereaved Dementia Caregivers. This project was conducted with community members and university members serving as Co-Principal Investigators. Community PIs lead a grassroots organization that has served Bereaved Dementia Caregivers since 2016. The University PI leads a research team devoted to understanding and improving the experience of Bereaved Dementia Caregivers. The present symposium will discuss the challenges and opportunities offered by this unique collaboration and insights gleaned by a project designed to serve doubly underrepresented groups: African American Bereaved Dementia Caregivers (who sometimes call themselves the “ultra-invisible”). Because this was a project funded through grant dollars, we will also discuss actionable insights that we hope may help others develop fruitful academic-community partnerships. Finally, we will share recruitment strategies for speaking with minoritized populations and insights from engaging gleaned through conversations with African American Bereaved Dementia Caregivers.

